# Lipoic Acid Exacerbates Oxidative Stress and Lipid Accumulation in the Liver of Wistar Rats Fed a Hypercaloric Choline-Deficient Diet

**DOI:** 10.3390/nu13061999

**Published:** 2021-06-10

**Authors:** Lidia V. Kravchenko, Ilya V. Aksenov, Nikolay S. Nikitin, Galina V. Guseva, Ludmila I. Avrenyeva, Nikita V. Trusov, Anastasia S. Balakina, Victor A. Tutelyan

**Affiliations:** 1 Federal Research Centre of Nutrition and Biotechnology, 2/14 Ustinsky Passage, 109240 Moscow, Russia; l.v.kravch@mail.ru (L.V.K.); nikolay_sergeevich87@mail.ru (N.S.N.); mailbox@ion.ru (G.V.G.); avrenyeva@ion.ru (L.I.A.); nikkitosu@yandex.ru (N.V.T.); balakina.a.s@yandex.ru (A.S.B.); tutelyan@ion.ru (V.A.T.); 2 FSAEI HE I.M. Sechenov First MSMU MOH Russia (Sechenovskiy University), 8-2 Trubetskaya Street, 119991 Moscow, Russia

**Keywords:** non-alcoholic fatty liver disease, α-lipoic acid, hypercaloric choline-deficient diet, oxidative stress, liver lipid accumulation

## Abstract

Non-alcoholic fatty liver disease (NAFLD) is currently estimated as the most prevalent chronic liver disease in all age groups. An increasing body of evidence obtained in experimental and clinical data indicates that oxidative stress is the most important pathogenic factor in the development of NAFLD. The study aimed to investigate the impact of α-lipoic acid (LA), widely used as an antioxidant, on the effects of a hypercaloric choline-deficient diet. Male Wistar rats were divided into three groups: control diet (C); hypercaloric choline-deficient diet (HCCD), and hypercaloric choline-deficient diet with α-lipoic acid (HCCD+LA). Supplementation of HCCD with LA for eight weeks led to a decrease in visceral adipose tissue/body weight ratio, the activity of liver glutathione peroxidase and paraoxonase-1, plasma, and liver total antioxidant activity, as well as an increase in liver/body weight ratio, liver total lipid and triglyceride content, and liver transaminase activities compared to the HCCD group without LA. In conclusion, our study shows that α-lipoic acid detains obesity development but exacerbates the severity of diet-induced oxidative stress and lipid accumulation in the liver of male Wistar rats fed a hypercaloric choline-deficient diet.

## 1. Introduction

Non-alcoholic fatty liver disease (NAFLD) is strongly associated with obesity and type 2 diabetes, and is now recognized as the most prevalent chronic liver disease in all age groups. It is estimated that about one-fourth of the global population and 90% of individuals with morbid obesity have NAFLD [1,2]. However, the pathogenesis of NAFLD progression from the stage of simple steatosis, characterized by liver fat accumulation, to non-alcoholic steatohepatitis, additionally characterized by persistent liver inflammation, balloon dystrophy of hepatocytes, and fibrosis, is still not fully elucidated [3,4].

An increasing body of evidence obtained in experimental and clinical data indicates that oxidative stress is the most important pathogenic factor in the development of NAFLD. Numerous researchers have noted a correlation between the severity of NAFLD and the intensity of oxidative stress [3,5,6,7]. Mitochondria are considered to be the main source of reactive oxygen species (ROS) in NAFLD. It is believed that excessive accumulation of lipids in the liver leads to mitochondrial ultrastructural damage and electron chain impairment and results in increased lipid peroxidation and production of ROS, which further promotes oxidative stress and inflammation [6,8,9]. A certain contribution to the generation of oxidative stress in NAFLD is made by the microsomal enzyme cytochrome P450 2E1 (CYP2E1), which is a potential producer of free radicals, H_2_O_2_, and a lipid peroxidation initiator [10]. NAFLD is accompanied by an increase in the CYP2E1 gene and protein expression, the accumulation of lipid peroxides in the liver [11]. Reactive oxidants are counterbalanced by a complex antioxidant defense system, which is assessed mainly by the activity of key antioxidant enzymes—superoxide dismutase (SOD), catalase (CAT), glutathione peroxidase (GPx), and the ratio of reduced (GSH) and oxidized (GSSG) glutathione in the liver. SOD maintains the intracellular content of superoxide radical at a low level, converting it into less active H_2_O_2_. CAT and GPx detoxify H_2_O_2_ and lipid peroxides. The results of several clinical and experimental studies indicate a direct relationship between the suppression of antioxidant enzyme activities and the severity of NAFLD [12,13,14,15]. At the same time, it was experimentally shown that the activation of the nuclear factor erythroid 2 (NF-E2)-related factor 2 (Nrf2), the main regulator of the expression of antioxidant enzyme genes [16], leads to suppression of oxidative stress and a decrease in liver inflammation and fibrosis [17]. The establishment of the pathogenetic role of oxidative stress in the progression of NAFLD has prompted an intensive study of the different classes of antioxidants as preventive and therapeutic agents for this disease [18,19].

The study aimed to investigate the impact of α-lipoic acid (LA), which is widely used as an antioxidant [20], on the effects of hypercaloric choline-deficient (HCCD) diet in rats.

## 2. Materials and Methods

### 2.1. Experimental Protocol

Male Wistar rats were obtained from the “Stolbovaya” nursery of the FMBA Scientific Center for Biomedical Technologies (Stolbovaya Settlement, Chekhov District, Moscow Region, Russian Federation). The study was approved by the Ethics Committee of the Federal Research Center of Nutrition and Biotechnology (No. 7/24.06.2019) and was performed following the European Convention for the Protection of Vertebrate Animals used for Experimental and Other Scientific Purposes.

Animals were divided into three groups (*n* = 8 in each): control pair-feeding group of rats fed a control diet (C) (Table 1); rats fed a hypercaloric choline-deficient diet (HCCD); rats fed a hypercaloric choline-deficient diet supplemented with α-lipoic acid (LA) (Chem Impex International, Inc., Wood Dale, USA, Cat# 29862) (HCCD+LA; consumed average daily dose of LA-61 mg/kg body weight).

During the 8 consecutive weeks, rats had free access to water and HCCD (20 g/day). Pair-feeding has been used in animals with the control diet by the following formula. The mean food intake in the HCCD group was calculated every week. The next week, the same amount of control diet was provided to the paired animals. Rats were maintained in plastic cages (2 animals in each) in a conditioned room (12-h light/dark cycle). Body weight was measured weekly; food intake was measured 5 times per week. Calorie intake was calculated by food intake (g) × energy density (kcal); feed efficiency ratio by body weight gain (g) / total food consumed (g).

At the end of the experimental period, the animals were subjected to 16–18 h of fasting. After decapitation blood was collected in tubes with heparin (Greiner Bio-One GmbH, Kremsmünster, Austria, Item No. 455051), and visceral (epididymal and retroperitoneal) adipose tissue (VAT) and liver were isolated. Plasma was obtained by centrifugation of blood samples at 3000 rpm for 20 min. Liver homogenates, cytosolic and microsomal fractions were prepared according to Lake (1987) [21].

### 2.2. Metabolic Measurements

Plasma alanine aminotransferase (ALT), aspartate aminotransferase (AST), glucose, triglycerides (TG), total cholesterol (T-Chol), high-density (HDL-Chol) and low-density (LDL-Chol) lipoprotein cholesterol, and free fatty acids (FFA) were analyzed by automated biochemical equipment Konelab 20i (Thermo Clinical Labsystems Oy, Espoo, Finland).

### 2.3. Liver Lipids Content

Total liver lipids were extracted and measured according to Folch (1957) [23]. Liver triglycerides and cholesterol content was analyzed using automated biochemical equipment Konelab 20i (Thermo Clinical Labsystems Oy, Espoo, Finland) after the dissolution of extracted lipids in ethanol (95% solution).

### 2.4. Total Antioxidant Activity (AOA)

The FRAP (Ferric Reducing Antioxidant Power) test system [24] was used to determine AOA. Briefly, 60 μL of plasma or cytosolic fraction of liver was added to 1.80 mL of the reaction mixture, consisting of 0.83 mM 2,4,6-tripyridyl-s-triazine and 1.67 mM FeCl_3_ × 6H_2_O in 0.25 M acetate buffer (pH 3.6) with subsequent measurement of the change in absorption at 593 nm for 4 min.

### 2.5. Glutathione Content

The glutathione content was assessed according to Anderson (1985) [25]. The liver tissue (0.5 g) was homogenized in 2.5 mL of 5% sulfosalicylic acid at 1200 rpm for 90 s. The homogenate (1.6 mL) was centrifuged at 10,000× *g* and 4 °C for 5 min and 0.25 mL of the supernatant was mixed with 12 μL of distilled water (for total glutathione determination) or vinylpyridine (for oxidized glutathione (GSSG) determination), and 0.375 mL of 0.5 M phosphate buffer (pH 7.5), followed incubation in the dark for 1 h. Next, 0.1 mL of the mixture was added to the reaction mixture, which consisted of 6 mL of 0.18% ethylenediaminetetraacetic acid disodium salt dihydrate (EDTA-Na_2_), 1 mL of 0.16% β-nicotinamide adenine dinucleotide 2′-phosphate reduced tetrasodium salt hydrate (β-NADPH), 2 mL of 0.12% 5,5′-dithiobis (2-nitrobenzoic acid) (Ellman’s reagent) in ethanol, 2 μL of glutathione reductase (1.35 IU) with subsequent measurement of the change in absorption at 412 nm for 1 min. The reduced glutathione (GSH) content was determined by the difference between the level of total and oxidized glutathione.

### 2.6. CYP2E1 Activity

The CYP2E1 activity was assessed according to Carlson (1991) [26]. The reaction mixture (1 mL), which consisted of 0.05 M Tris base buffer (pH 7.4), 1 mM β-NADPH, 5 mM MgCl_2_, 0.2 mM p-nitrophenol, liver microsomal fraction (~3 mg of protein), was incubated at 37 °C within 15 min. Next, 0.25 mL of 0.6 N HClO_4_ was added and the mixture was centrifuged at 3500 rpm for 10 min. The supernatant (1 mL) was mixed with 10 N NaOH (0.1 mL) with subsequent measurement of absorbance at 546 nm. The enzyme activity was calculated using the molar extinction coefficient of p-nitrophenol ε = 9.53 mM^−1^ cm^−1^.

### 2.7. Antioxidant Enzyme Activities

The paraoxonase-1 (PON-1) activity was determined according to Beltowski et al. (2005) [27]. To the reaction mixture (final volume 3 mL), which consisted of 0.1 M Tris base-HCl buffer (pH 8.0), 2 mM CaCl_2_, 2 mM phenylacetate were added 10 μL of plasma or microsomal fraction of liver (~0.08 mg of protein) with subsequent measurement of the change in absorption at 270 nm for 3 min at 25 °C. The enzyme activity was calculated using the molar extinction coefficient of phenylacetate ε = 1310 M^−1^ cm^−1^.

The superoxide dismutase (SOD) activity was measured according to Nishikimi et al. (1972) [28]. The reaction mixture consisted of 1.25 mL of 2 mM sodium pyrophosphate buffer, 2 mM EDTA-Na_2_, 50 μL of the cytosolic fraction of liver (~0.8 mg of protein), 170 µL of 0.5 mM nitro blue tetrazolium, 170 µL of 1.4 mM β-nicotinamide adenine dinucleotide, reduced disodium salt hydrate, and 170 µL of 22.2 μM phenazine methosulfate. The enzyme activity was assessed by measurement of the change in absorption at 540 nm for 1 min at 20 °C. SOD activity was expressed in units (1 U = 50% inhibition of formazan formation) per mg of protein.

The catalase (CAT) activity was assessed according to Aebi (1984) [29]. The reaction mixture consisted of 2.43 mL of 0.1 M phosphate buffer (pH 7.0), 1 mM EDTA-Na_2_, 1 mM NaN_3_, 0.1 mL of the cytosolic fraction of liver (~1.6 mg of protein), and 12.5 μL of 10% H_2_O_2_. The enzyme activity was assessed by measurement of the change in absorption at 240 nm for 1 min at 20 °C and calculated using the molar extinction coefficient of H_2_O_2_ ε = 0.0394 mM^−1^ cm^−1^.

The glutathione peroxidase (GPx) activity was determined according to Flohé and Günzler (1984) [30]. The reaction mixture consisted of 2.375 mL of 0.1 M phosphate buffer (pH 7.0), 1 mM EDTA-Na_2_, 1 mM NaN_3_, 25 μL of the cytosolic fraction of liver (~0.4 mg of protein), 50 μL of 50 mM GSH, 50 μL of 9.6 mM β-NADPH in 1% NaHCO_3_, 0.74 IU of glutathione reductase, 5 μL of 0.64% H_2_O_2_ in C_2_H_5_OH. The enzyme activity was assessed by measurement of the change in absorption at 340 nm for 1 min at 30 °C and calculated using the molar extinction coefficient of β-NADPH ε = 6220 M^−1^ cm^−1^.

### 2.8. Histopathological Examination

The pieces of the liver large left lobe were fixed in 10% neutral-buffered-formalin for 3 days, embedded in paraffin by an automatic tissue processing machine (Donatello, Diapath S.p.A., Martinengo, Italy), and sectioned at 4 µm slices with a microtome (Microm HM-355S, Thermo Scientific, Germany). The sections were stained with hematoxylin-eosin and van Gieson stains according to the standard procedure [31], visualized using a light microscope (Axio Imager Z1, Carl Zeiss Microscopy GmbH, Göttingen, Germany) at ×100 magnification, photographed by a digital camera (AxioCam HRc, Carl Zeiss Microscopy GmbH, Göttingen, Germany), and evaluated for severity of lipid infiltration, lobular inflammation, ballooning degeneration, and fibrosis using program software (AxioVision Rel.4.8, Carl Zeiss Microscopy GmbH, Göttingen, Germany). The degree of liver lesions was assessed using the semiquantitative scoring system SAF (steatosis, activity, fibrosis) [32].

### 2.9. Statistical Analysis

Kruskal–Wallis test followed by the Dunn’s multiple comparisons test was used to compare the severity of NAFLD in liver samples. Other obtained results were analyzed using one-way analysis of variance (ANOVA) followed by the Tukey post hoc test. Data are expressed as mean ± standard error of the mean. Significance was established at a value of *p* < 0.05.

## 3. Results

### 3.1. Weight Characteristics and Nutritional Profile

As shown in Table 2 the initial body weight of rats of all three groups did not differ. At the end of the experimental period, the body weight of the rats fed HCCD exceeded that in the control group by 14%. The supplementation of HCCD with LA led to an insignificant decrease in the final body weight of rats in comparison to rats fed HCCD without LA. Rats fed HCCD gained more body weight (by 39%) compared to those fed the control diet. There was no difference between HCCD and HCCD+LA groups for body weight gain.

**Figure 1:**
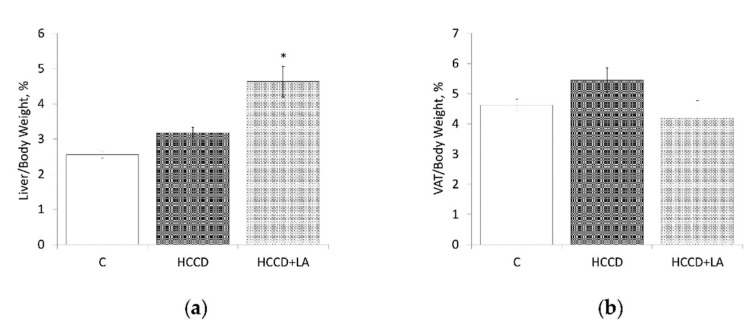
Effects of α-lipoic acid (LA) supplementation on: (**a**) liver/body weight ratio; (**b**) visceral adipose tissue (VAT)/body weight ratio. Animals were divided into three groups: control group of rats fed a control diet (C); rats fed a hypercaloric choline-deficient diet (HCCD); rats fed a hypercaloric choline-deficient diet supplemented with α-lipoic acid (HCCD+LA; consumed average daily dose of LA-61 mg/kg body weight). VAT: visceral (epididymal and retroperitoneal) adipose tissue. Values are mean ± standard error of the mean. *p* < 0.05 vs. * C.

Food consumption did not significantly change over the experimental period between the groups studied. At the same time, daily calorie intake in HCCD and HCCD+LA groups of rats exceeded that in the control group by 28% and 27%, respectively; feed efficiency ratio-by 37% and 31%. Ratio liver/body weight was higher (by 26%) in the rats fed HCCD in comparison to the control group (HCCD: 3.17 ± 0.16 vs. C: 2.52 ± 0.09%) (Figure 1).

The supplementation of HCCD with LA led to a significant increase in liver/body weight ratio (to 4.63 ± 0.44%), which was 46% higher than in rats fed HCCD without LA and 84% more than in the control group of animals. Ratio VAT/body weight was higher (by 18%) in the rats fed HCCD in comparison to the control group (HCCD: 5.45 ± 0.41 vs. C: 4.62 ± 0.20%). The supplementation of HCCD with LA led to a significant decrease in VAT/body weight ratio (to 4.19 ± 0.50%), which was even lower (by 10%) than in rats fed the control diet.

### 3.2. Liver Lipid Content

It was showed a significant increase in the content of total lipids (threefold), triglycerides (TG) (eightfold), and total cholesterol (T-Chol) (threefold) in the liver of rats fed HCCD in comparison to the control group (Table 3). The supplementation of HCCD with LA led to an additional increase in the liver content of total lipids (by 45%), TG (by 54%), and total cholesterol (by 30%) in comparison to the HCCD group without LA.

### 3.3. Metabolic Parameters

**Figure 2:**
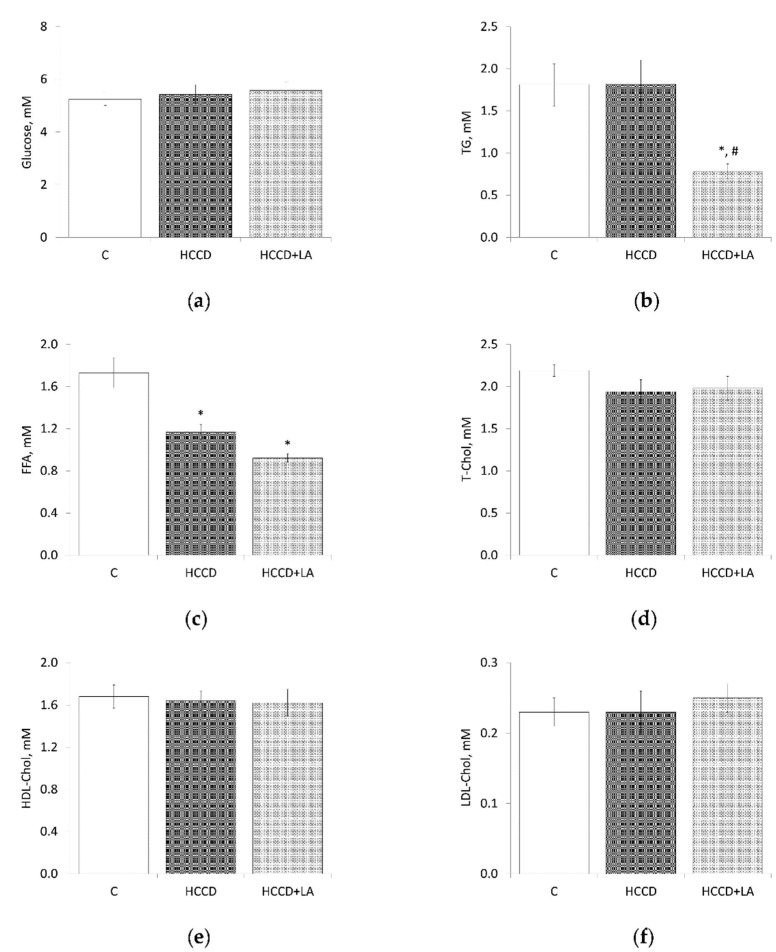
Effects of α-lipoic acid (LA) supplementation on plasma levels of (**a**) glucose, (**b**) triglycerides (TG), (**c**) free fatty acids (FFA), (**d**) total cholesterol (T-Chol), (**e**) high-density lipoprotein cholesterol (HDL-Chol), (**f**) low-density lipoprotein cholesterol (LDL-Chol). Animals were divided into three groups: control group of rats fed a control diet (C); rats fed a hypercaloric choline-deficient diet (HCCD); rats fed a hypercaloric choline-deficient diet supplemented with α-lipoic acid (HCCD+LA; consumed average daily dose of LA-61 mg/kg body weight). Values are mean ± standard error of the mean. *p* < 0.05 vs. * C; ^#^ HCCD+LA vs. HCCD.

The consumption of the HCCD diet for eight weeks did not cause significant changes in the plasma concentration (mM) of glucose (HCCD: 5.42 ± 0.37 vs. C: 5.25 ± 0.24), TG (HCCD: 1.82 ± 0.28 vs. C: 1.81 ± 0.25), T-Chol (HCCD: 1.94 ± 0.14 vs. C: 2.19 ± 0.07), HDL-Chol (HCCD: 1.64 ± 0.09 vs. C: 1.68 ± 0.11), LDL-Chol (HCCD: 0.23 ± 0.03 vs. C: 0.23 ± 0.02) (Figure 2). At the same time, the rats of this group showed a significant (by 32%) decrease in the plasma FFA content (HCCD: 1.17 ± 0.07 vs. C: 1.73 ± 0.14 mM). The supplementation of the HCCD with LA did not affect the plasma level of glucose, T-Chol, HDL-Chol, and LDL-Chol, but was accompanied by a significant, more than twofold, decrease in the concentration of TG-to 0.78 ± 0.09 mM. Also, LA caused a decrease in the plasma FFA level to 0.92 ± 0.04 mM, which was lower than in rats fed HCCD without LA and in rats fed the control diet by 21% and 47%, respectively.

### 3.4. Oxidative Stress Markers

**Figure 3:**
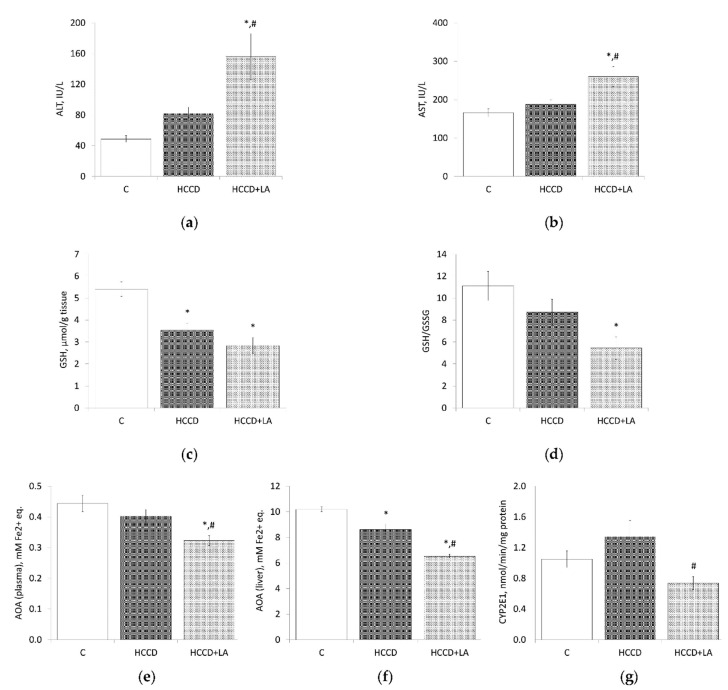
Effects of α-lipoic acid (LA) supplementation on (**a**) alanine aminotransferase (ALT) activity in plasma; (**b**) aspartate aminotransferase (AST) activity in plasma; (**c**) reduced glutathione (GSH) level in the liver; (**d**) reduced/oxidized glutathione ratio (GSH/GSSG) in the liver; (**e**) total antioxidant (AOA) activity in plasma; (**f**) total antioxidant (AOA) activity in the liver; (**g**) cytochrome P450 2E1 (CYP2E1) activity in the liver. Animals were divided into three groups: control group of rats fed a control diet (C); rats fed a hypercaloric choline-deficient diet (HCCD); rats fed a hypercaloric choline-deficient diet supplemented with α-lipoic acid (HCCD+LA; consumed average daily dose of LA-61 mg/kg body weight). Values are mean ± standard error of the mean. *p* < 0.05 vs. * C; ^#^ HCCD+LA vs. HCCD.

In the plasma of rats fed HCCD diet a 68% increase in ALT activity (HCCD: 81.8 ± 8.6 vs. C: 48.8 ± 4.5 IU/L) and a 13% increase in AST activity (HCCD: 188 ± 12 vs. C: 166 ± 10 IU/L) was observed (Figure 3). The concentration of GSH in the liver of the HCCD group decreased by 35% (HCCD: 3.54 ± 0.31 vs. C: 5.41 ± 0.33 μmol/g tissue). At the same time, the liver GSH/GSSG ratio decreased by 22% (HCCD: 8.7 ± 1.2 vs. C: 11.1 ± 1.3%). AOA was slightly lower in the plasma of rats fed HCCD in comparison to the control group (HCCD: 0.40 ± 0.02 vs. C: 0.44 ± 0.03 mM Fe^2+^-equivalents), but significantly decreased (by 16%) in the cytosolic fraction of the liver (HCCD: 8.6 ± 0.4 vs. C: 10.2 ± 0.2 mM Fe^2+^-equivalents). The activity of microsomal CYP2E1 in the liver of rats in the HCCD group exceeds that in the control group by 22% (HCCD: 1.34 ± 0.21 vs. C: 1.05 ± 0.11 nmol/min/mg protein).

Treatment with LA led to a significant increase in plasma ALT activity (156 ± 30 IU/L), in comparison to the control group (threefold) and rats fed HCCD without LA (twofold). LA had a similar effect on plasma AST activity, which increased by 57% compared to the control group and by 38%-to the HCCD group without LA. The GSH content (2.84 ± 0.36 μmol/g tissue) in the liver of rats treated with LA was 48% lower than in the control group, and 20% lower than in the HCCD group without LA. The supplementation with LA led to a significant change in the GSH/GSSG ratio in the liver, reducing it to 5.5 ± 1.0%, which was 50% compared to the control group, and 63%-to the group of rats fed HCCD without LA. In rats treated with LA, a decrease in the AOA of both blood plasma and the cytosolic fraction of the liver was observed. Thus, AOA in plasma (0.32 ± 0.02 mM Fe^2+^-equivalents) and liver (6.5 ± 0.1 mM Fe^2+^-equivalents) was lower, respectively, by 37% and 36%, than in the control group and by 20% and 24% compared to the group of rats fed HCCD without LA. Noteworthy is the 45% inhibition of CYP2E1 activity in the liver of rats treated with LA, compared with rats fed HCCD without LA.

**Figure 4:**
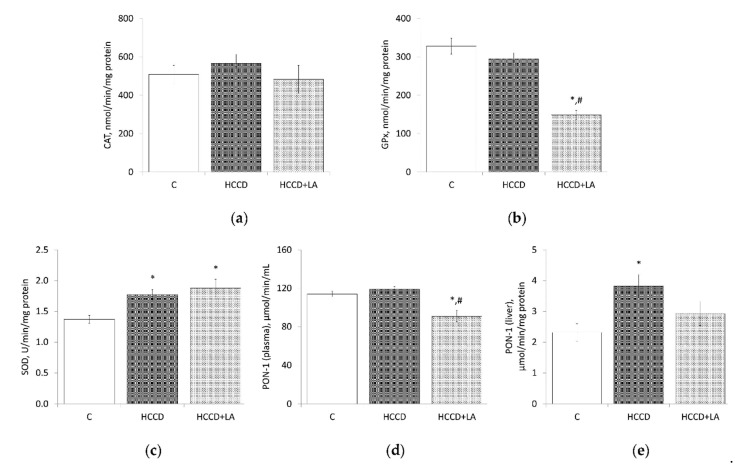
Effects of α-lipoic acid (LA) supplementation on (**a**) catalase (CAT) activity in the liver; (**b**) glutathione peroxidase (GPx) activity in the liver; (**c**) superoxide dismutase (SOD) activity in the liver; (**d**) paraoxonase-1 (PON-1) activity in plasma; (**e**) paraoxonase-1 (PON-1) activity in the liver. Animals were divided into three groups: control group of rats fed a control diet (C); rats fed a hypercaloric choline-deficient diet (HCCD); rats fed a hypercaloric choline-deficient diet supplemented with α-lipoic acid (HCCD+LA; consumed average daily dose of LA-61 mg/kg body weight). Values are mean ± standard error of the mean. *p* < 0.05 vs. * C; ^#^ HCCD+LA vs. HCCD.

As shown in Figure 4, HCCD did not affect CAT and GPx activities but led to an increase in SOD activity in the liver by 29% compared to the control group (HCCD: 1.77 ± 0.09 vs. C: 1.37 ± 0.07 U/min/mg protein).

There was no HCCD-related change in the activity of PON-1 in plasma, but an increase in enzyme activity in the liver of rats treated with HCCD by 65% (HCCD: 3.82 ± 0.38 vs. C: 2.31 ± 0.28 μmol/min/mg protein) should be noted. The LA supplementation led to an additional increase in the liver SOD activity by 37% (up to 1.88 ± 0.14 U/min/mg protein) compared to the control group, a decrease in CAT activity by 15% (HCCD: 566 ± 48 vs. HCCD+LA: 483 ± 72 nmol/min/mg protein) and a deep suppression of GPx activity-by 49% (HCCD: 295 ± 16 vs. HCCD+LA: 149 ± 12 nmol/min/mg protein), in comparison to the rats fed HCCD without LA. LA decreased PON-1 activity by 24% both in plasma (up to 91 ± 6 μmol/min/mL) and in the liver (up to 2.92 ± 0.39 μmol/min/mg protein) compared to the rats fed HCCD without LA.

### 3.5. Histopathological Examination

**Figure 5:**
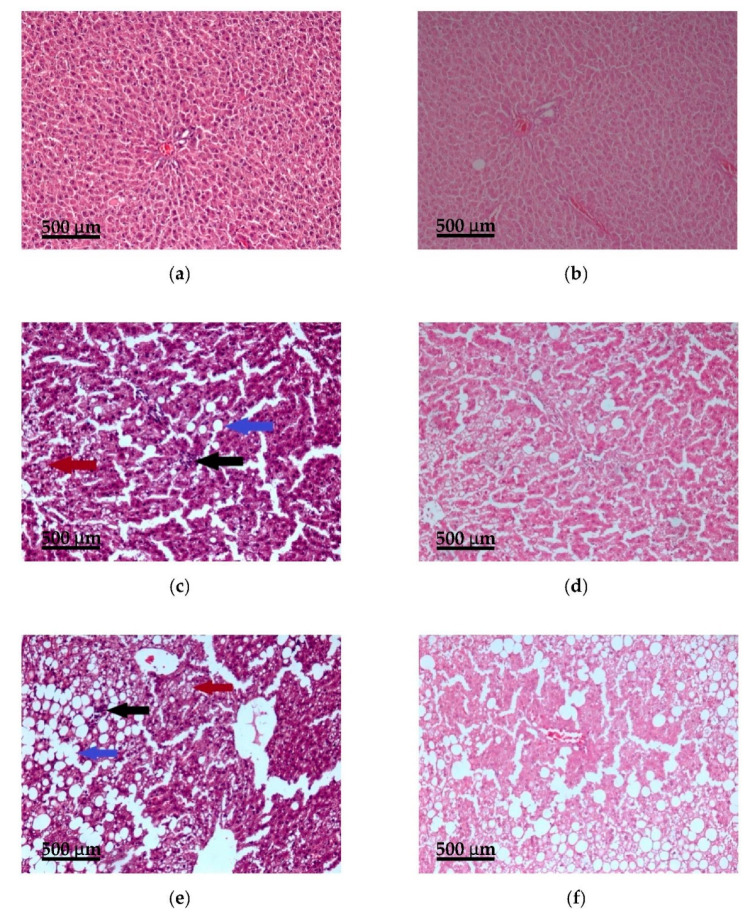
Effects of α-lipoic acid (LA) supplementation on histological features of the liver (portal area, ×100): (**a**) control group, hematoxylin-eosin stain; (**b**) control group, van Gieson stain; (**c**) hypercaloric choline-deficient diet group, hematoxylin-eosin stain; (**d**) hypercaloric choline-deficient diet group, van Gieson stain; (**e**) hypercaloric choline-deficient diet supplemented with α-lipoic acid (consumed average daily dose of LA-61 mg/kg body weight), hematoxylin-eosin stain; (**f**) hypercaloric choline-deficient diet supplemented with α-lipoic acid (consumed average daily dose of LA-61 mg/kg body weight), van Gieson stain. Large-sized lipid droplets are indicated by the blue arrow, foci of lobular inflammation by the black arrow, and hepatocellular ballooning by the red arrow. Scale bar = 500 μm.

Histopathological examination showed a predominantly normal liver architecture in the control group (eight liver samples) (Figure 5). Hepatocytes had a cuboidal shape, sharp angles, and pink eosinophilic cytoplasm; the nuclei were in the center of the cells, no lobular inflammation or periportal and centrilobular fibrosis were observed. In only two liver samples was observed mild steatosis: small and medium-sized lipid droplets were revealed in 5–20% of hepatocytes. The median SAF score in the control group was S0A0F0 (healthy liver).

In all seven liver samples from rats fed HCCD, medium and large-sized lipid droplets (in 15–70% of hepatocytes) and lobular inflammation (less than two foci per lobule) without fibrosis were detected. Hepatocellular ballooning (the clusters of hepatocytes with a rounded shape and pale usually reticulated cytoplasm and one or several enlarged hepatocytes) was detected in six samples. The median SAF score in the HCCD group was S1A3F0 (steatohepatitis).

LA supplementation led to statistically insignificant changes in the liver compared to the HCCD group: an increase in lipid accumulation (all eight liver samples contained large-sized lipid droplets in 30–90% of hepatocytes), a decrease in hepatocellular ballooning (was detected in four samples) and in lobular inflammation (was observed in six samples). In one liver sample bridging fibrosis was detected. The median SAF score in the HCCD+LA group was S2A2F0 (steatohepatitis).

## 4. Discussion

In our study, Wistar rats fed a hypercaloric choline-deficient diet with an excess amount of lard (42% of total diet calories) and fructose (20% of total diet calories). Investigation of the effects of high-fat diets with different sources of fat on the development of obesity and steatosis in Wistar rats showed that lard led to metabolic changes most characteristic for obesity and to steatosis without signs of inflammation and clear fibrotic phenomena in the liver [33].

The results obtained in our work showed that feeding of rats with HCCD for eight weeks leads to an increase in the final body weight (by 14%) and weight gain as a result of a higher calorie intake and an increase in feed efficiency. This coincides with the data of other authors, according to which, Wistar rats fed a high-fat diet with lard had higher final body weight by 10–15% than those of the control [34,35,36]. Moreover, it was shown that the concomitant excess of carbohydrates (sucrose) in the diet did not affect the elevation in body weight [36].

The moderate increase in liver weight in rats fed HCCD was associated with significant lipid accumulation in the liver, which is one of the early manifestations of NAFLD. Similar changes in the liver induced by high-fat and hypercaloric diets have been described earlier [8,36,37,38].

The assessment of visceral fat accumulation was recommended earlier as a good estimate for obesity in the rat [39]. In our study, we found a moderate, by 18%, increase in the VAT/body weight ratio and by 35% in the absolute weight of visceral fat in rats fed HCCD for eight weeks. Similar changes—an increase in adipose tissue/body weight ratio by 17%, were found earlier in Wistar rats fed a high-fat diet with lard for four weeks [37]. With a longer, for 20 weeks, feeding Wistar rats with a diet with a high content of lard and sucrose, the weight of visceral fat exceeded the control level by 39% [36]. It should be noted that dietary choline levels can also affect obesity. In most cases, choline-deficient diets did not lead to significant obesity [4].

Dyslipidemia is considered one of the hallmarks of NAFLD, but it has been shown that its severity can vary significantly or even be absent [14,15,35,38,40].

Our findings showed that feeding rats with HCCD did not lead to pronounced changes in the level of glucose, total cholesterol, HDL- and LDL-cholesterol, triglycerides in the blood, which is consistent with other studies in Wistar rats fed high-fat diets with lard or hypercaloric diets with lard and sucrose [34,35,36,38]. As for the decrease in the level of free fatty acids in the blood observed in our study, the literature indicates the possibility of both increasing and decreasing their level using diets with lard [34].

Several signs of oxidative stress were found in rats fed HCCD, including an increase in the activity of ALT and AST in the blood, which is the most frequently detected marker of NAFLD and other liver damage. Another important indicator of the severity of oxidative stress in rats of this group was a decrease (by 35%) in the level of GSH, the main intracellular antioxidant, and a decrease (by 22%) in the GSH/GSSG ratio in the liver. HCCD led to a decrease in the total antioxidant activity of the liver and, to a lesser extent, blood plasma, which also indicated the presence of oxidative stress [7]. The detected activation of CYP2E1, although it was not statistically significant, suggests that hydroperoxides and lipid peroxides formed with the participation of CYP2E1 are involved in the development of oxidative stress in rats fed HCCD. It has been shown that in mice with NAFLD, induction of CYP2E1 is accompanied by a 100-fold increase in the content of lipid peroxides in the liver [11]. At the same time, the antioxidant quercetin prevents the development of NAFLD in mice fed a high-fat diet by the suppression of the CYP2E1 gene expression and CYP2E1-dependent lipid peroxidation [41].

The imbalance between pro-oxidants and antioxidants and the severity of oxidative stress largely depends on the activity of antioxidant enzymes such as SOD, CAT, and GPx. SOD maintains a low intracellular concentration of highly active superoxide radical, catalyzing its dismutation to O_2_ and less active hydroperoxide H_2_O_2_, which undergoes further detoxification with the participation of CAT and GPx. Analysis of the role of individual antioxidant enzymes (CAT and GPx) in oxidative stress in NAFLD led the researchers to the assumption that excessive H_2_O_2_ production may be an important event triggering NAFLD [9,42].

PON-1 plays an important role in antioxidant defense, protecting lipoproteins and cell membranes from oxidative modification. PON-1 is mainly synthesized by the liver and secreted bound to HDL into the circulation [43,44]. This enzyme shares the same regulatory pathways as other antioxidant enzymes [45]. PON-1 activity in plasma correlated with the severity of oxidative stress and lipid accumulation in the liver, which made it possible to consider the observed suppression of PON-1 activity as one of the signs of liver damage [46,47].

The scientific literature regarding the activity of antioxidant enzymes in NAFLD is contradictory. In rats with high-fat diet-induced NAFLD, a decrease in SOD, CAT, GPx, and PON-1 activities in the liver and serum was detected [13,38]. Besides, a relationship between the suppression of PON-1 activity and the severity of NAFLD was noted. In the study with mice fed a high-fat diet and fructose, the activities of SOD, CAT, and GPx increased in the early stages of the experiment and decreased later [5]. Patients with NAFLD had higher antioxidant enzyme activities (SOD, GPx, and CAT) in the liver cytosol whereas activities of those enzymes in erythrocytes did not differ compared to control [48]. However, it has also been shown an increase in erythrocyte SOD activity without difference in GPx activity in patients with NAFLD compared to healthy controls [7].

In our study, feeding rats with HCCD for eight weeks led to a substantial (by 65%) activation of PON-1, a moderate increase (by 29%) in SOD activity without a significant change in the activities of CAT and GPx in the liver in comparison to the control group. The imbalance between SOD and GPx activity can lead to the accumulation of H_2_O_2_ and oxidative stress aggravation [42].

It should be noted that ROS are activators of the transcription factor Nrf2, which regulates the initiation of gene expression of antioxidant enzymes and thus can indirectly activate Nrf2-related enzymes [16]. Therefore, the selective activation of SOD and PON-1 can be considered as a compensatory mechanism for redox imbalance in the liver.

Thus, feeding Wistar rats with HCCD for eight weeks led to moderate obesity (with an increase in visceral fat mass) and NAFLD (steatohepatitis) without severe dyslipidemia (except a decrease in the plasma level of free fatty acids). At the same time, several oxidative stress hallmarks were found—an increase in hepatic transaminase activities, a decrease in the GSH content and activation of microsomal CYP2E1 in the liver, a decrease in plasma and liver total antioxidant activity. Selective compensatory activation of certain antioxidant enzymes confirms moderate-intensity oxidative stress.

LA is considered a compound able to detain the development of obesity and NAFLD due to its antioxidant properties. In animal models of NAFLD, LA reduced obesity and lipid accumulation in the liver, suppressed the expression of CYP2E1 and H_2_O_2_ production, and restored the lowered activity of antioxidant enzymes [37,38,49,50].

In our study, the supplementation of HCCD with LA at a daily dose of 61 mg/kg body weight for eight weeks did not significantly affect the feed efficiency and final body weight but decreased the visceral adipose tissue/body weight ratio to a level below the control. The ability of LA to reduce obesity and body weight was shown in several studies [51,52]. It is believed that LA exerts anti-obesity effects by suppressing hypothalamic AMP-activated protein kinase activity followed by a reduction in food intake and an increase in energy expenditure [53,54]. Zucker diabetic fatty rats fed LA showed a decrease in epididymal fat weight per body weight ratio associated with an upregulation of epididymal uncoupled protein-1 that plays a key role in non-shivering thermogenesis [55].

In contrast to the data of Seo et al. [37], in our experiment, the treatment with LA led to a significant increase (by 46%) in the liver/body weight ratio compared to rats fed HCCD without LA. This was accompanied by an increase (by 45%) in the content of total lipids and more expressed accumulation of large-sized lipid droplets in the rat liver which indicates an increase in the severity of HCCD-induced lipid accumulation under the impact of LA.

The supplementation of HCCD with LA more than halved the plasma concentration of triglycerides, which is a characteristic action of LA in obesity [56] and enhanced the inhibitory effect of HCCD on the plasma level of FFA.

The progression in lipid accumulation in the liver of rats fed LA correlated with the severity of oxidative stress: a significant increase in ALT and AST plasma activity, a decrease in GSH content, and, to an even greater extent, the GSH/GSSG ratio in the liver, a decrease in plasma and liver total antioxidant activity. Also, an imbalance was observed between an increase in SOD activity and a twofold decrease in the activity of GPx, which could lead to the accumulation of H_2_O_2_ and the subsequent formation of a highly aggressive hydroxyl radical [42]. Enhanced H_2_O_2_ production was detected in the liver of rats fed a high-fat diet for eight weeks [38]; high level of H_2_O_2_ in the blood of patients with NAFLD [57].

Suppression of the CYP2E1 activity, as well as the activity of antioxidant enzymes such as GPx, CAT, PON-1 in rats fed LA, might be the result of a direct action of ROS on the enzyme protein or a consequence of damage to hepatocytes and disruption of the enzyme synthesis.

A few experimental studies devoted to assessing the safety of long-term prophylactic treatment with LA, indicate that it can exhibit pro-oxidant properties, cause oxidative stress, disrupt lipid metabolism and lead to liver steatosis [58,59,60]. Thus, in C57BL6/J mice treated with LA at a dose of 20 mg/kg body weight for four weeks was shown development of oxidative stress: an increase in the content of malondialdehyde in blood and 8-OH-dG in the liver, a decrease in the GSH/GSSG ratio in the liver. Long-term (for 74 weeks) LA supplementation caused an increase in lipogenesis in the liver. LA led to an increase in the plasma triglycerides content and liver cholesterol, the development of liver steatosis, the most pronounced, including with foci of necrosis, with prolonged treatment [58]. In another study, the increased levels of protein oxidation markers after LA administration were considered as a result of the pro-oxidant effects of LA [59].

In conclusion, our study shows that α-lipoic acid detains obesity development but exacerbates the severity of diet-induced oxidative stress and lipid accumulation in the liver of male Wistar rats fed a hypercaloric choline-deficient diet.

## Figures and Tables

**Table 1:** Composition of diets.

Ingredient(g/kg Diet)	Diets	
	C	HCCD
Casein (>85% protein)	140	140
Cornstarch	721.2	271.2
Fructose	-	246
Sunflower Oil	20	20
Lard	20	226
Fiber (Cellulose Powder)	50	50
Mineral Mix (AIN-93M-MX) [22]	35	35
Vitamin Mix (AIN-93-VX) [22]	10	10
Choline Chloride (52% choline)	2.0	-
L-Cysteine	1.8	1.8
Energy Density (kcal/g)	3.89	4.92

Composition of the control diet (C) and the hypercaloric choline-deficient diet (HCCD) is presented. Formulation of the Mineral Mix and the Vitamin Mix correspond to those (named in parenthesis) recommended by the American Institute of Nutrition (AIN) for maintenance of adult rodents [22]. “-”—Was not added to the diet.

**Table 2:** Effects of α-lipoic acid (LA) supplementation on body weight and nutritional profile.

Parameter	C	HCCD	HCCD+LA
Initial Body Weight, g	215 ± 1	207 ± 5	204 ± 6
Final Body Weight, g	364 ± 5	414 ± 13 *	404 ± 20
Body Weight Gain, g	149 ± 5	207 ± 14 *	199 ± 19 *
Total Food Consumed, g	870 ± 21	881 ± 27	882 ± 10
Food Intake, g/day	16.0 ± 0.4	16.3 ± 0.5	16.0 ± 0
Calorie Intake, kcal/day	62.3 ± 1.8	79.5 ± 2.6 *	79.3 ± 1.1 *
Feed Efficiency Ratio	0.172 ± 0.006	0.235 ± 0.017	0.226 ± 0.028

Animals were divided into three groups: control group of rats fed a control diet (C); rats fed a hypercaloric choline-deficient diet (HCCD); rats fed a hypercaloric choline-deficient diet supplemented with α-lipoic acid (HCCD+LA; consumed average daily dose of LA-61 mg/kg body weight). Calorie intake was calculated by food intake (g) × energy density (kcal); feed efficiency ratio-by body weight gain (g)/total food consumed (g). Values are mean ± standard error of the mean. *p* < 0.05 vs. * C.

**Table 3:** Effects of α-lipoic acid (LA) supplementation on liver lipid content.

Parameter (mg/g Liver Tissue)	C	HCCD	HCCD+LA
Total Lipids	70 ± 3	237 ± 21 *	343 ± 21 *^,#^
TG	17 ± 2	140 ± 18 *	215 ± 19 *^,#^
T-Chol	3.3 ± 0.4	9.6 ± 1.1 *	12.5 ± 1.1 *

Animals were divided into three groups: control group of rats fed a control diet (C); rats fed a hypercaloric choline-deficient diet (HCCD); rats fed a hypercaloric choline-deficient diet supplemented with α-lipoic acid (HCCD+LA; consumed average daily dose of LA-61 mg/kg body weight). TG: triglycerides; T-Chol: total cholesterol. Values are mean ± standard error of the mean. *p* < 0.05 vs. * C; ^#^ HCCD+LA vs. HCCD.

## Data Availability

The data presented in this study are available on request from the corresponding author.
